# Analysis of the Effect of Velocity on the Eddy Current Effect of Metal Particles of Different Materials in Inductive Bridges

**DOI:** 10.3390/s22093406

**Published:** 2022-04-29

**Authors:** Wei Li, Shuang Yu, Hongpeng Zhang, Xingming Zhang, Chenzhao Bai, Haotian Shi, Yucai Xie, Chengjie Wang, Zhiwei Xu, Lin Zeng, Yuqing Sun

**Affiliations:** 1 School of Marine Engineering, Dalian Maritime University, Dalian 116026, China; dmuliwei@dlmu.edu.cn (W.L.); yush312@163.com (S.Y.); zhxm@hit.edu.cn (X.Z.); baichenz@163.com (C.B.); dmu6hao@163.com (H.S.); xyc86418332@163.com (Y.X.); wangcj@dlmu.edu.cn (C.W.); xuzhiwei201809@163.com (Z.X.); sunyq@dlmu.edu.cn (Y.S.); 2 School of Naval Architecture and Ocean Engineering, Harbin Institute of Technology, Weihai 264209, China; 3 Laboratory of Biomedical Microsystems and Nano Devices, Bionic Sensing and Intelligence Center, Institute of Biomedical and Health Engineering, Shenzhen Institute of Advanced Technology, Chinese Academy of Sciences, Shenzhen 518055, China; lin.zeng@siat.ac.cn

**Keywords:** inductance bridge, particle material, velocity, eddy current effect, oil detection

## Abstract

A method for analyzing the influence of velocity changes on metal signals of different materials in oil detection technology is proposed. The flow rate of metal contaminants in the oil will have a certain impact on the sensitivity of the output particle signal in terms of electromagnetic fields and circuits. The detection velocity is not only related to the sensitivity of the output particle signal, but also to the adaptability of high-speed and high-throughput in oil online monitoring. In this paper, based on a high-sensitivity inductive bridge, the eddy current effect of velocity in a time-harmonic magnetic field is theoretically analyzed and experimentally verified, the phenomenon of particle signal variation with velocity for different materials is analyzed and discussed, and finally the effect of velocity on the output signal of the processing circuit is also elaborated and experimentally verified. Experiments show that under the influence of the time-harmonic magnetic field, the increase of the velocity enhances the detection sensitivity of non-ferromagnetic metal particles and weakens the detection sensitivity of non-ferromagnetic particles. Under the influence of the processing circuit, different velocities will produce different signal gains, which will affect the stability of the signal at different velocities.

## 1. Introduction

The Real Economy is the foundation of a country’s economic development, involving industry, agriculture, construction, and other fields. The operation stability of various heavy machinery in the real economy determines the engineering efficiency and quality. Therefore, mechanical fault diagnosis is directly related to the economic cost of engineering and indirectly related to the development of the country’s real economy. In the context of such engineering needs, the fault diagnosis of mechanical equipment shows its criticality. Fault diagnosis is a state monitoring technology for mechanical equipment during operation. By grasping the noise [1], vibration [2], temperature [3], oil quality [4,5], and other information of mechanical equipment during operation, it is possible to realize the type judgment of mechanical faults, fault location, and early prediction of mechanical faults [6]. There are four main means of fault diagnosis techniques [7]: noise detection, vibration detection, temperature detection, and oil detection. Among them, the oil detection technology [8,9] is widely used in engineering because it can detect various information in the mechanical system, so as to obtain more comprehensive fault information such as mechanical wear degree, fault type, and fault location. The detected information includes oil viscosity [10], air bubbles [11], water content [12], and metal particle concentration [13,14], size [15], type [16] in the oil.

However, there are still problems of the low sensitivity of large-throughput detection and low-throughput detection which is not conducive to online detection [17]. Solving the contradiction has become the research direction of researchers in related fields. In 2009, Murali S et al. [18] designed a microfluidic oil detection chip based on the principle of capacitive Coulter counting, which combined the microfluidic chip with the oil detection technology, greatly reducing the size of the microfluidic oil detection chip and improving the sensitivity of metal particle detection in oil. In 2011, Du L et al. [19] designed a microfluidic oil detection chip based on the inductance Coulter principle, which can realize the differentiated identification of ferrous metals and non-ferrous metals. Zhang X et al. [20] studied the detection mechanism of the inductive oil detection chip, established a system model between the detection coil and metal particles, and obtained the mathematical model between the number of turns, density, excitation frequency, metal particle size, and output inductance of the detection coil. Respectively, Ma L et al. [21], Li Y et al. [22] used the mutual inductance between double coils and the optimization of triple coil structure to improve the detection sensitivity of the sensor. Davis J P et al. [23] realized the high-sensitivity identification of metal particles under the condition of the simplified circuit by using Maxwell bridge and subsequent filtering, amplifying, and rectifying circuits. As mentioned above, many researchers have been working to achieve high-sensitivity differential detection from various aspects, including structural design [24], circuit design [25], magnetic material addition [26]. To further realize online monitoring of mechanical faults and expand the detection throughput with high detection sensitivity, a series of studies have been carried out by the researchers concerned. Zhu X et al. [27] adopted the time-division multiplexing method to collect the sensing data of 9 channels in sequence, and added series diodes to eliminate the crosstalk between each channel. Jagtiani A V et al. [28] adopted the frequency division multiplexing method to modulate and demodulate multiple signals with different frequency carriers, increasing the detection throughput by 4 times. Bai C et al. [29] designed a high-throughput sensor with an annular flow channel structure, expanding the flow to 16 times that of the original microchannel.

Other related researchers started from the perspective of detection speed, explored the possibility of online monitoring of oil, and explored the impact of speed on detection sensitivity. Wang X et al. [30] explored the influence of particle velocity on the aliasing signal waveform, and put forward the conclusion that the aliasing induced voltage is proportional to the particle velocity. Wu Y et al. [31] deduced the analytical formula for the influence of the flow rate on the magnetic field, and proposed that the detection sensitivity of copper particles can be enhanced by increasing the oil detection flow rate. Liu E et al. [32] studied the influence of flow velocity on the detection signal, and proposed that the amplitude of the inductive signal of metal particles can be improved by appropriately reducing the flow velocity. The research results of the above researchers are valuable, but there is still controversy about the effect of flow rate on abrasive particles of different materials.

In this study, based on an inductive bridge, we explore the effect of particle velocities of different materials on the eddy current effect. The influence of particle motion velocity on the output signal of detected particles is analyzed by using the angle difference between the direction of particle motion and the direction of magnetic field lines on both sides of the coil. The characteristics of high-speed and high throughput regard online monitoring are fitted under the premise of high detection sensitivity. Compared with previous analysis of velocity, this method can visually verify the trend of particle velocity to signal change without interference from electromagnetic and detection circuits. In this paper, an inductive bridge and a processing circuit are used to replace the impedance analyzer, so the sensor still has high detection sensitivity under the premise of portability and low cost. And the content explored in this paper is in line with the actual engineering, which is conducive to the development of online monitoring. On this basis, it is committed to the realization of online detection of oil, by exploring the influence of speed on the detection of metal particles of different materials.

## 2. Sensor Design and Theory Analysis

### 2.1. Inductance Detection Analysis

**Figure 1:**
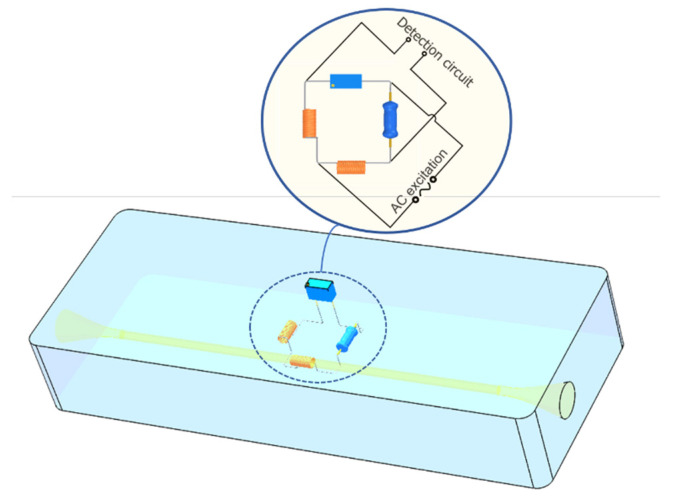
Schematic diagram of microfluidic chip sensing unit.

The design of the sensor chip is shown in Figure 1. The inside of the sensing chip includes the particle inlet and outlet, the detection channel, and the inductive bridge. In the inductive bridge, one of the spiral coils wrapped around the outside of the detection channel is the induction coil and the other spiral coil is the balance coil. Fixed resistance, variable resistance and two solenoid coils form an electric bridge, and the inductive pulses generated by particles passing through the induction coils are converted into differential voltage pulses by the inductive bridge. As shown in Figure 1, the two nodes on the bridge were connected to the AC excitation, and the voltage signals output by the other two nodes were connected to the subsequent detection circuit.

According to previous studies [33], the amount of inductance change produced by the particle at the central axis of the solenoid coil is:

(1) ΔLx=Im(Δzmaxω)=4πμ0N2w2+d2Re(kp)

where $\Delta z_{\max}$ is the impedance output by the coil detecting the particle, ω is the angular frequency of the AC excitation, $\mu_{0}$ is the vacuum permeability, N is the number of turns of the coil, w is the axial length of the coil, and d is the inner diameter of the coil.

The magnetization factor $k_{p}$ of ferromagnetic metal particles is:

(2) $$k_{p}=\frac{\left(-r^{2}k^{2}+2\mu_{r}+1\right)\sin\left(rk\right)-rk\left(2\mu_{r}+1\right)\cos\left(rk\right)}{\left(r^{2}k^{2}+\mu_{r}-1\right)\sin\left(rk\right)-rk\left(\mu_{r}-1\right)\cos\left(rk\right)}\cdot \frac{r^{3}}{2}$$

The magnetization factor $k_{p}$ of the non-ferromagnetic metal particles is:

(3) $$k_{p}=\frac{1}{2}\cdot \left[r^{3}+\frac{3r^{2}}{k}\cot\left(rk\right)-\frac{3r}{k^{2}}\right]$$

where r is the particle radius and $\mu_{r}$ is the relative magnetic permeability of the particle, which is assumed to be a real number in this paper and is not affected by frequency.

(4) $$k=\sqrt{-j\omega \mu_{r}\mu_{0}\sigma }$$

where σ is the electrical conductivity.

When an AC excitation is applied to the coil, an alternating magnetic field is generated inside the coil. Since the relative permeability of ferromagnetic metal particles is much greater than 1, the magnetization effect generated when the ferromagnetic metal particles pass through the induction coil causes the magnetic flux in the coil to increase abruptly. However, the induced current is generated inside the ferromagnetic metal particles to obstruct the change of the original magnetic field, and the induced current generates magnetic flux in the opposite direction of the original magnetic field, which weakens the original magnetic field. That is, the particles produce eddy current effect in the time-harmonic magnetic field. For ferromagnetic particles, the magnetization effect is stronger than the eddy current effect, so the amount of change in coil inductance caused by ferromagnetic metal particles is positive.

Since the relative permeability of non-ferromagnetic metal particles is slightly less than 1, the eddy current effect dominates when the non-ferromagnetic metal particles pass through the induction coil, causing the original magnetic field to be weakened, so the change in coil inductance caused by the non-ferromagnetic metal particles is negative. The sensor can differentiate and detect ferromagnetic and non-ferromagnetic metal particles in the oil, based on the Coulter principle. The tribological information reflected by the detected metal particle information (including the number of metal particles obtained by the number of inductance signals, and the particle size of the metal particles obtained by the amplitude of the inductance signal) can be used to predict the degree of failure of the system.

### 2.2. Design and Principle Analysis of Inductor Bridge

**Figure 2:**
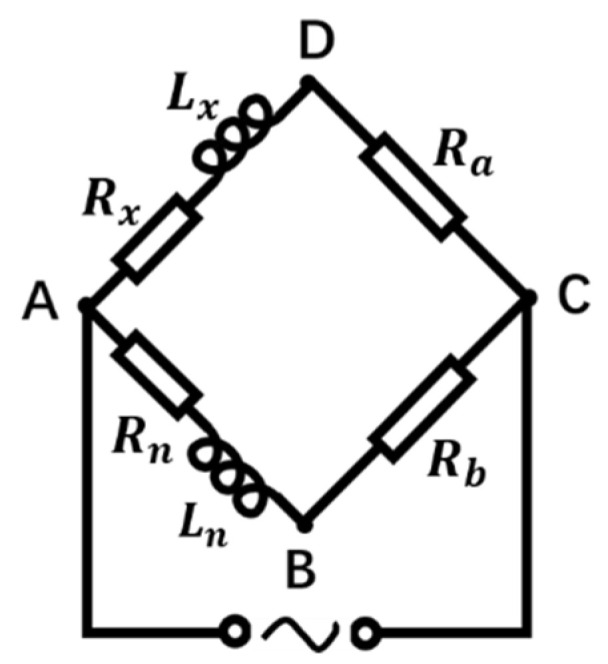
Inductive bridge equivalent circuit.

Considering the bridge as an equivalent circuit, the equivalent circuit is shown in Figure 2 A, B, C, D are the endpoints on the bridge arms; $L_{x}$ is the inductance of the induction coil; $R_{x}$ is the internal resistance of the induction coil; $L_{n}$ is the inductance of the reference coil; $R_{n}$ is the internal resistance of the reference coil; $R_{a}$ and $R_{b}$ are the balance resistances on the two bridge arms, respectively.

**Figure 3:**
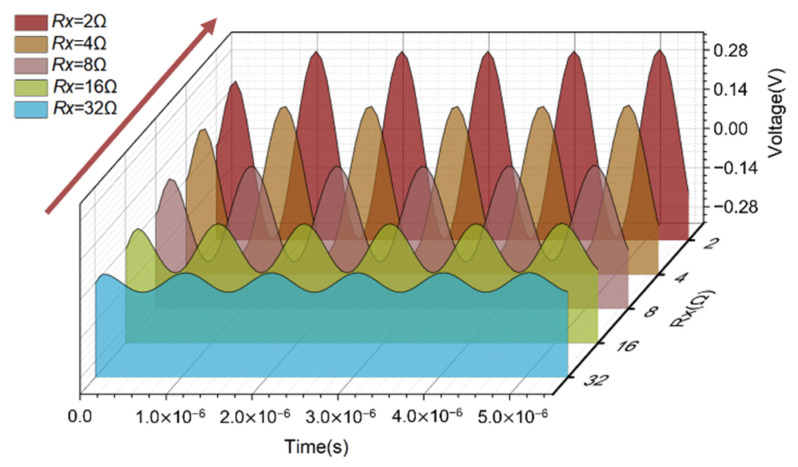
Effect of coil quality factor on bridge sensitivity.

Simulate the equivalent circuit of the inductive bridge. The frequency of the AC excitation source was set to 1 MHz, and the voltage was set to 10 V. Explore the effect of resistance parameters on bridge sensitivity. First, change the resistance values of $R_{a}$ and $R_{b}$ at the same time; second, change the resistance values of the coil internal resistance $R_{x}$ and $R_{n}$ at the same time. Under the condition that the inductance base value of the two coils $L_{x}$, $L_{n}$ was 1 μH and the inductance change $\Delta L_{x}$ was 0.1 μH, the change of the coil internal resistance had a great influence on the voltage difference $U_{BD}$ between the two points B and D. As shown in Figure 3, the direction of the arrows shows the trend of bridge sensitivity as a function of internal resistance. As the coil internal resistance $R_{x}$ decreased, the voltage value of $U_{BD}$ increased under the condition of the same inductance variation $\Delta L_{x}$, namely, the higher the quality factor of the coil, the higher the sensitivity of the bridge. Combined with the analysis of the simulation results, it is expected to select the coil with a smaller internal resistance $R_{x}$ under the condition of similar base inductance $L_{x}$.

**Figure 4:**
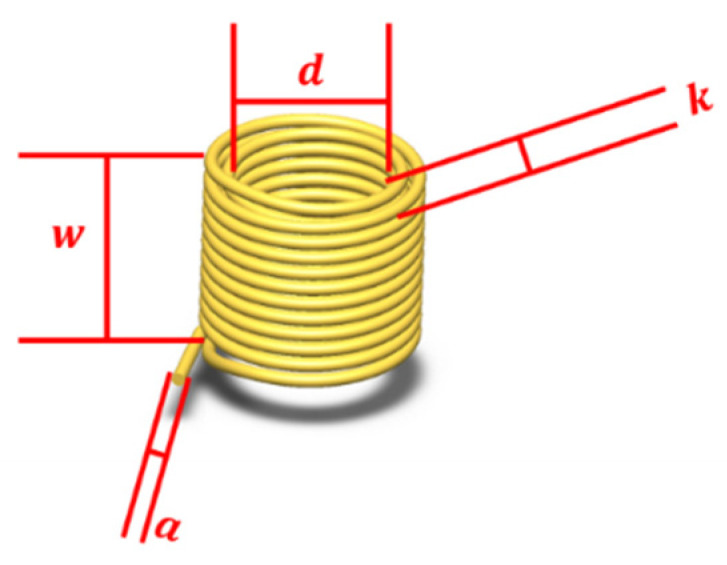
Schematic diagram of coil parameters.

As shown in Figure 4, w is the height of the coil; k is the number of layers of the coil; a is the wire diameter of the coil; d is the inner diameter of the coil.

The expression for the amount of change in coil inductance $\Delta L_{x}$ due to particles in Equation (1) shows that the amount of change in inductance $\Delta L_{x}$ is related to the coil turns N, axial length w, coil inner diameter d, particle radius r, relative permeability of particles $\mu_{r}$, particle conductivity σ, and the angular frequency ω of the AC excitation applied to the coil. Among them, the factors affected by the coil itself are: the number of turns of the coil N, the axial length w, and the inner diameter of the coil d. Let $b=\frac{N^{2}}{w^{2}+d^{2}}$. The larger b is, the larger the change in coil inductance $\Delta L_{x}$ is, and the higher the sensitivity to detect particles as they pass through the coil. Since $\frac{\partial b}{\partial N}>0$, $\frac{\partial b}{\partial w}<0$, and $\frac{\partial b}{\partial d}<0$, it means that the larger the number of coil turns N, the smaller the coil axis length w and the inner diameter d, the higher the coil inductance variation $\Delta L_{x}$. In order to produce a large inductance variation $\Delta L_{x}$ between the metal particles and the coil, it is desired to increase the number of turns N of the coil, while being able to reduce the coil axis length w and the inner diameter d.

The expression for the estimated internal resistance of the spiral coil is:

(5) $$R_{x}=\rho \cdot \frac{l}{S}=\rho \cdot \frac{2\pi \cdot \frac{N}{k}\cdot \sum_{i=0}^{k=i+1}\left\{\left[a\left(2i+1\right)+d\right]/2\right\}}{\pi \cdot {\left(\frac{a}{2}\right)}^{2}}$$

where ρ is the coil resistivity, l is the coil length, and S is the coil cross-sectional area.

It can be seen from Equation (5) that the larger the wire diameter a that determines the cross-sectional area of the coil S, the smaller the number of turns N, the axial length w, and the inner diameter d that collectively reflect the coil length l, the smaller the coil internal resistance $R_{x}$. Therefore, increasing the wire diameter of the coil a can reduce the internal resistance of the coil $R_{x}$ without affecting the variation of the coil inductance $\Delta L_{x}$.

Since the microfluidic chip adopts tiny coils, the wire diameter a which is smaller than the number of coil turns N, axial length w and inner diameter d is regarded as a fixed value, and the part as the numerator is ignored in the calculation of the partial derivative between the axial length w and the number of coil turns N. Then there is $\frac{\partial R}{\partial w}>0$, $\frac{\partial R}{\partial d}>0$, $\frac{\partial R}{\partial N}>0$, that is, the coil resistance $R_{x}$ decreases monotonically with the decrease of the number of turns N, axial length w, and inner diameter d. Since the wire diameter a is much smaller than the number of coil layers k, the axial length w and the inner diameter d, namely, $\frac{\partial R}{\partial N}<\frac{\partial R}{\partial w}$ and $\frac{\partial R}{\partial N}<\frac{\partial R}{\partial d}$, which means the change rate of the coil resistance $R_{x}$ with the number of turns N is smaller than the change rate of the resistance $R_{x}$ with the axial length w and inner diameter d. Collectively, the coil resistance $R_{x}$ can be reduced by decreasing the number of turns N, axial length w, and inner diameter d. The effect of a change in the number of turns N is less than the effect of a change in the axial length w and inner diameter d on the resistance $R_{x}$. Therefore, the number of turns N of the coil can be increased, while the resistance $R_{x}$ can be appropriately sacrificed, and the coil inductance variation $\Delta L_{x}$ can be increased at the same time.

To summarize, in order to select a coil with smaller resistance $R_{x}$ under the condition of larger inductance change $\Delta L_{x}$, a larger coil wire diameter a, smaller axial length w, inner diameter d, and appropriately larger number of turns N can be selected.

Combined with the above theoretical analysis and debugging through experiments, the number of turns N = 230 turns, axial length w = 2.76 mm, inner diameter d = 0.54 mm, outer diameter D = 1.55 mm, wire diameter a = 0.07 mm, resistance $R_{x}$ = 4 Ω of the solenoid coil were selected by the bridge sensing unit.

When the particles pass through the coil to generate an inductive signal, the two arms of the bridge generate a voltage difference [34], the voltage of the two arms are rectified into two DC pulsating voltage signals, and then filtered by the filter circuit to remove the high frequency noise signal [35], interspersed in the particle signal, and finally differentially amplified by an amplifier circuit to generate the differential voltage caused by the particles.

**Figure 5:**
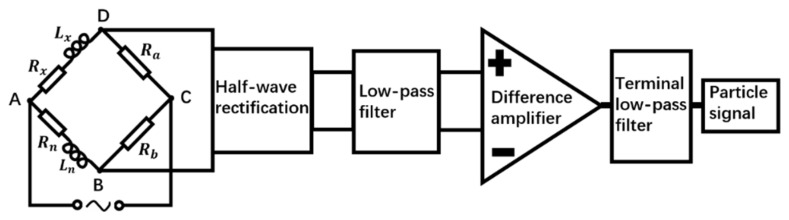
Schematic diagram of detection circuit system.

As shown in the block diagram of the detection circuit system in Figure 5, when the bridge is balanced:

(6) $$Z_{AB}\cdot Z_{CD}=Z_{AD}\cdot Z_{BC}$$

That is:

(7) $$\left(j\omega L_{x}+R_{x}\right)\cdot R_{b}=\left(j\omega L_{n}+R_{n}\right)\cdot R_{a}$$

(8) $$U_{BD}=U_{AD}-U_{AB}=U_{i}\cdot \left(\frac{Z_{AD}}{Z_{AD}+Z_{CD}}-\frac{Z_{AB}}{Z_{AB}+Z_{BC}}\right)$$

(9) $$U_{BD}=U_{i}\{\frac{\omega^{2}[L_{x}^{2}R_{b}\left(R_{n}+R_{b}\right)-L_{n}^{2}R_{a}\left(R_{x}+R_{a}\right)]+\left(R_{n}+R_{b}\right)\left(R_{x}+R_{a}\right)\left(R_{x}R_{b}-R_{n}R_{a}\right)}{\left[\omega^{2}L_{x}^{2}+{\left(R_{x}+R_{a}\right)}^{2}\right]\left[\omega^{2}L_{n}^{2}+{\left(R_{n}+R_{b}\right)}^{2}\right]}+\frac{\omega L_{x}R_{a}\left[\omega^{2}L_{n}^{2}+{\left(R_{n}+R_{b}\right)}^{2}\right]-\omega L_{n}R_{b}\left[\omega^{2}L_{x}^{2}+{\left(R_{x}+R_{a}\right)}^{2}\right]}{\left[\omega^{2}L_{x}^{2}+{\left(R_{x}+R_{a}\right)}^{2}\right]\left[\omega^{2}L_{n}^{2}+{\left(R_{n}+R_{b}\right)}^{2}\right]}\cdot j\}$$

When ferromagnetic metal particles pass through an alternating time-harmonic magnetic field in the inductive bridge, the inductance value of the induction coil increases, resulting in an increase in $U_{BD}$, and the differential voltage is positive; when the non-ferromagnetic metal particles pass through the inductive bridge, the inductance value of the induction coil decreases, resulting in a decrease in $U_{BD}$, the differential voltage is negative.

In Figure 5, a half-wave rectifier circuit module using diodes was used to rectify two AC voltage signals into pulsating DC voltage signals respectively. Since the amplitude and direction of the DC signal do not change with time, the rectified pulsating DC voltage signal can realize the differential detection of ferromagnetic and non-ferromagnetic metal particles by an inductive bridge, and can be better collected and compared.

In the low-pass filter module using the two-stage wireless gain multiple feedback filter circuit, the cut-off frequency of the first-stage low-pass filter circuit was 16 kHz, and the cut-off frequency of the second-stage low-pass filter circuit was 1.6 kHz. The low-pass filter module can reduce the AC components in the two-channel pulsating DC voltage signals as much as possible, and filter out the high-frequency noise and harmonics mixed in the two-channel signals.

The differential amplifier module made the voltage difference between the two signals affected by the sensing coil and the reference coil, and the differential voltage signal combined with the reverse amplifier circuit could be amplified by 20 to 400 times.

In the previous experimental research in the laboratory, the DC signal generated by the AC voltage signal through the above-mentioned rectification, filtering and amplifying circuit still has relatively large noise. Therefore, the terminal filter circuit was adopted in the laboratory to further extract the particle signal with very low frequency. The terminal filter using the UAF42 active filter can adjust the cutoff frequency of the low-pass filter in the range of 0~5 kHz and the quality factor of the filter by changing the resistance value of the potentiometer.

The high-sensitivity detection of ferromagnetic and non-ferromagnetic metal particles in oil detection and the portability of the detection device are realized through the processing of the inductive bridge and the detection circuit.

### 2.3. The Effect of Velocity on the Magnetic Field of a Solenoid

The velocity of metal particles in the sensing coil is a non-negligible factor affecting the particle signal. The velocity of the metal particles passing through the sensing coil affects both the sensing signal [36] and the flux of the oil detection.

According to Faraday’s law of electromagnetic induction:

(10) $$\varepsilon =-\frac{d\Phi }{dt}$$

where ε is the induced electromotive force generated by the particle passing through the coil, and $\frac{d\Phi }{dt}$ is the rate of change of the magnetic flux with time.

According to earlier research in our lab [21]:

(11) $$\varepsilon =\oint_{C}{\vec{E}}_{in}\cdot dl=-\frac{d\Phi }{dt}=-\frac{d}{dt}\iint_{S}\vec{B}\cdot d\vec{S}=-\iint_{S}\left(\frac{\partial \vec{B}}{\partial t}\cdot d\vec{S}+\vec{B}\cdot \frac{\partial }{\partial t}d\vec{S}\right)$$

where ${\vec{E}}_{in}$ is the electric field strength of the metal particle induced by the magnetic field, dl is the length differential of the vortex ring inside the metal particle, $\vec{B}$ is the magnetic induction between the metal particle and the coil, $\vec{S}$ is the area of the vortex ring, $-\iint_{S}\frac{\partial \vec{B}}{\partial t}\cdot d\vec{S}$ is the induced electromotive force $\varepsilon_{t}$ due to the change in the magnetic field, and $-\iint_{S}\vec{B}\frac{\partial }{\partial t}\cdot d\vec{S}$ is the kinetic electromotive force $\varepsilon_{m}$ due to the movement of the metal particle in the magnetic field.

When the metal particles pass through the alternating magnetic field in the coil, an eddy current ring is generated inside the metal, and the resulting motional electromotive force is:

(12) $$\varepsilon_{m}=-\frac{d\phi_{m}}{dt}=\oint_{C}\left(\vec{v}\times \vec{B}\right)\cdot dl$$

where $\vec{v}$ is the moving velocity of the metal particles in the magnetic field.

According to the definition of inductance:

(13) $$dL=\frac{d\Phi }{dI}=\frac{\oint_{c}\left(\vec{v}\times \vec{B}\right)\cdot dl\;dt}{I}$$

It can be seen from Equation (13) that in the alternating magnetic field, the coil inductance dL caused by the motional electromotive force $\varepsilon_{m}$ increases with the increase of the velocity $\vec{v}$. That is, the greater the velocity, the greater the induced electromotive force containing the motional electromotive force, the stronger the obstruction of the original magnetic field by the induced electromotive force generated by the metal particles, and the more pronounced the eddy current effect inside the metal particles. This is reflected in the fact that, by increasing the velocity, the magnetization effect is weakened more by the eddy current effect in the detection of ferromagnetic metal particles, resulting in a weaker signal for ferromagnetic particles, while the eddy current effect is enhanced in the detection of non-ferromagnetic particles, resulting in a stronger signal for non-ferromagnetic particles.

## 3. Results and Discussion

**Figure 6:**
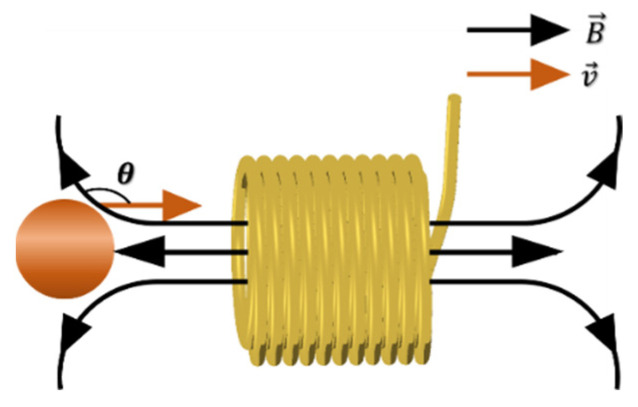
Schematic diagram of cutting magnetic field lines in the direction of movement of metal particles.

Experiments to explore the velocity often use a plane coil, so that the metal particles move on the surface of the plane coil, that is, in the direction perpendicular to the magnetic field lines of the plane coil. This method amplifies the proportion of the motional electromotive force in the induced electromotive force, which is ideal for the study of velocity effect. However, during the actual experiments, many uncontrollable factors arise at the same time as the velocity increases, such as the small sampling rate of the equipment, particle vibration and increased noise. These factors also affect the output of the signal. Using the angle difference θ between the movement direction of the metal particles on both sides of the solenoid coil and the magnetic field line inside the solenoid, as shown in Figure 6, the influence trend of the dynamic electromotive force can be clearly seen by comparison.

**Figure 7:**
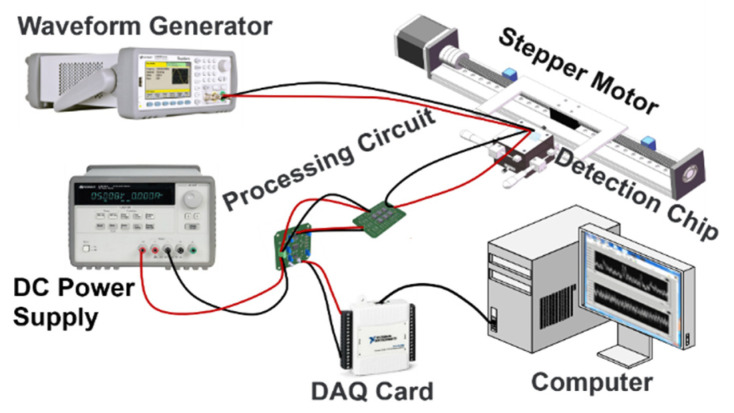
Experimental system diagram.

**Figure 8:**
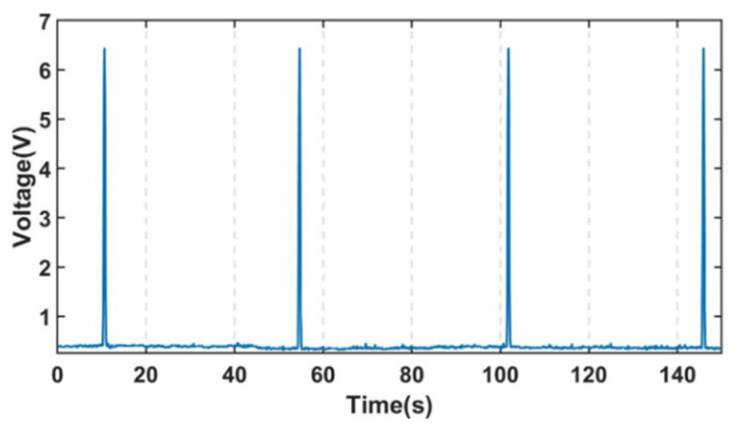
Steady sampling signal for 350 μm iron particles.

**Figure 9:**
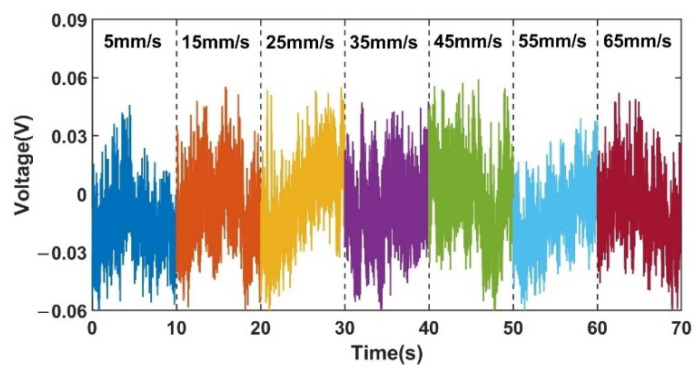
Noise signals at different velocities under no load.

In this experiment, the experimental system was set up as shown in Figure 7, with the metal particles adhering to a nylon rope and a stepper motor controlling the velocity past the detection chip. The particle inductance signal sensed by the sensing coil in the detection chip is converted into two voltage signals by the inductance bridge in the detection chip. When the metal particles on the nylon rope pass through the sensing coil, a voltage difference is generated between the two voltage signals. The inductive bridge in the detection chip was given a 1.3 MHz, 10 V sinusoidal AC excitation source by a waveform generator. The subsequent processing circuit rectifies, filters, differentiates and amplifies the two voltage signals generated at points B and D of the inductive bridge, and the processing circuit was powered by a DC power supply of ±15 V DC. The voltage signal processed by the processing circuit is converted by the data acquisition card to the computer through analog-digital conversion. To avoid distortion of the sampled signal when the sampling rate was insufficient, the sampling rate was chosen to be much greater than the signal frequency. The sampling rate chosen was 2000 samples/s, much greater than the frequency of the signal required by Nyquist-Shannon sampling theorem for this experiment. The experiment proved that the sampling signal at this sampling rate was stable, with a set of signals for 350 μm iron particles at a velocity of 5 mm/s as shown in Figure 8:

The experimental data of the acquired noise signal is shown in Figure 9. The noise signal is collected at each velocity for 10 s under the condition of no-load nylon rope. It can be seen in Figure 9 that the collected noise signal does not change much with the change of velocity.

**Figure 10:**
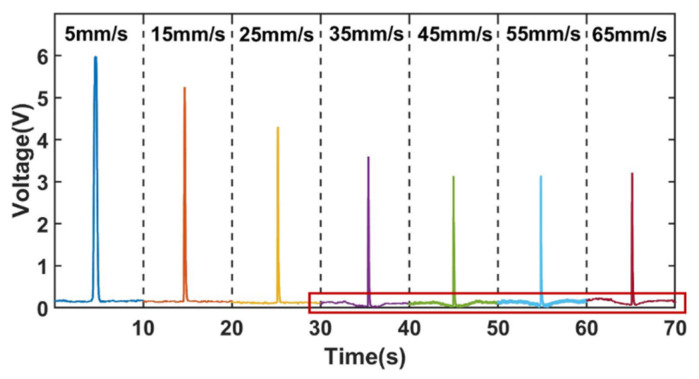
Comparison of signals at different velocities for 350 μm iron.

**Figure 11:**
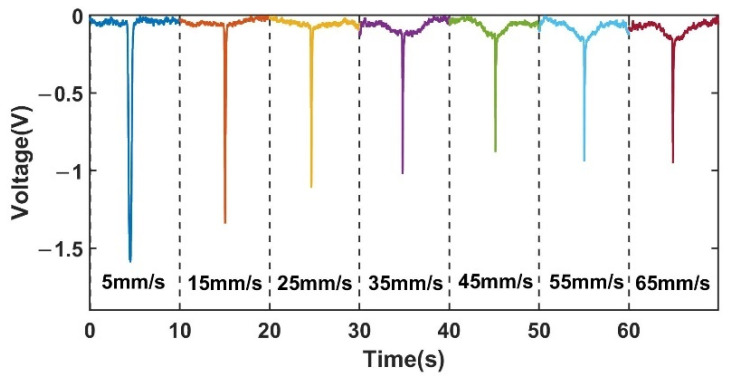
Comparison of signals at different velocities for 350 μm copper.

**Figure 12:**
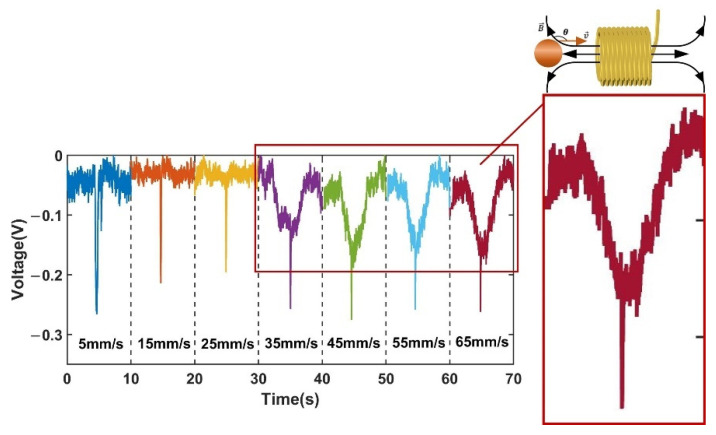
Comparison of signals at different velocities for 350 μm aluminum.

Figure 10 shows a comparison of the signals of 350 µm ferromagnetic metal particles (350 µm Fe) at different velocities, capturing one signal from a set at each velocity, which was intercepted for 10 s. Figure 11 shows a comparison of the signals of non-ferromagnetic metal particles (350 μm Cu) at different velocities, capturing one signal from a set at each velocity, which was intercepted for 10 s. Figure 12 shows a comparison of the signals of non-ferromagnetic metal particles (350 μm Al) at different velocities, capturing one signal from a set at each velocity, which was intercepted for 10 s.

As can be seen in Figure 10, Figure 11 and Figure 12, during the increase in velocity from 5 mm/s to 65 mm/s, the signal affected by the velocity of the cut magnetic induction lines before and after the spiral coil starts to increase significantly when the velocity increases to 35 mm/s. This phenomenon can be observed in the detection of iron particles, copper particles and aluminum particles. This phenomenon is caused by the angular difference θ between the moving direction of the particles and the direction of the alternating magnetic field. The change of velocity increases the induced electromotive force between the particles and the coil, and enhances the eddy current effect inside the particles. This phenomenon is consistent with the theoretical inference in 2.3.

**Figure 13:**
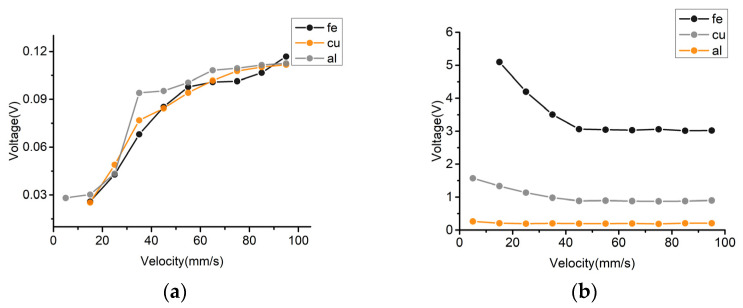
Diagram of signal variation with velocity: (**a**) Variation of metal particle voltage amplitude affected by velocity, (**b**) Variation of the total signal amplitude of the metal particles with varying velocity.

Figure 13a shows the changing trend of the voltage signal amplitude affected by the cutting magnetic field velocity for the iron, copper, and aluminum metal particles on both sides of the coil.

Although the eddy current effect caused by the enhanced induced electromotive force increases as the particle velocity increases, the amplitude of the voltage change caused by the velocity increases, resulting in a weakening of the signal of ferromagnetic metal particles and an enhancement of the signal of non-ferromagnetic metal particles. However, in general, the trend of the signal change of metal particles is still weakened, and the signal amplitude of non-ferromagnetic metal particles is still weakened. The signal weakening trend is shown in Figure 13b. The analysis of this phenomenon is presented as follows.

The increase of the detection velocity, on the one hand, for the time-harmonic magnetic field, changes the induced electromotive force between the metal particles and the coil in the magnetic field; on the other hand, for the detection circuit, it changes the frequency of the metal particle signal. That is, as the velocity of the metal particles increases, the signal frequency of the metal particles increases.

It can be seen by using the solenoid that in the time-harmonic magnetic field, the increase of the velocity leads to the enhancement of the induced electromotive force, which weakens the original magnetic induction intensity. This phenomenon is manifested as an increase in the negative detection signal caused by the induced electromotive force, which gradually increases with the increase of the detection velocity on both sides of the sensing area of the solenoid. This is reflected in the weakening of the detection signal of the ferromagnetic metal particles and the enhancement of the detection signal of the non-ferromagnetic metal particles. This experimental result is consistent with the theoretical inference in Section 2.3.

As for the non-magnetic factors during the experiment, that is, for the detection circuit, the change in the signal frequency of the metal particles causes a change in the signal output amplitude of the particles at the corresponding frequency components. According to the set stepper motor stroke, stepper motor acceleration time and the velocity of the particles passing through the sensing coil, it is known that when the particles pass through the sensing coil at a velocity of 5 mm/s, the particle signal frequency is 0.02 Hz; when the particle passes the sensing coil at a velocity of 15 mm/s, the frequency of the particle signal is 0.06 Hz; when the particle passes the sensing coil at a velocity of 25 mm/s, the frequency of the particle signal is 0.09 Hz; when the particle passes the sensing coil at a velocity of 35 mm/s, the frequency of the particle signal is 0.12 Hz; when the particle passes the sensing coil at a velocity of 45 mm/s, the frequency of the particle signal is 0.15 Hz; when the particle passes the sensing coil at a velocity of 55 mm/s, the frequency of the particle signal is 0.17 Hz; when the particle passes the sensing coil at a velocity of 65 mm/s, the frequency of the particle signal is 0.19 Hz. As can be seen, different detection velocities, will directly lead to changes in the frequency component of the acquired signal, and the change in frequency, which will further affect the effect of the processing of that frequency component in the detection circuit, namely, the change in the frequency component caused by the velocity enhancement, will directly lead to changes in the output signal of the detection circuit.

**Figure 14:**
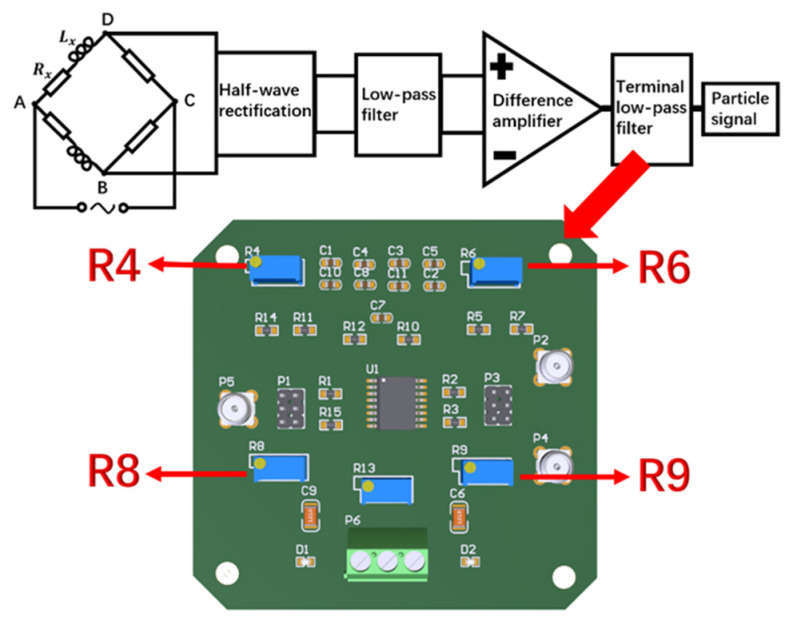
Terminal UAF42 low pass filter.

**Figure 15:**
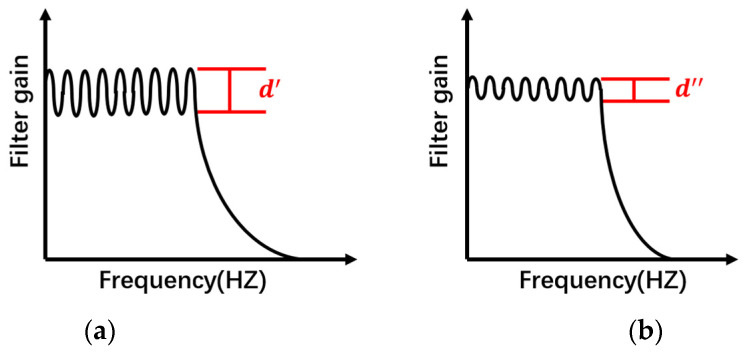
Characteristic curves of low-pass filter circuits with different design parameters: (**a**) Large passband ripple, (**b**) Small passband ripple.

As shown in Figure 14, the terminal filter selected in the detection circuit is taken as an example. For the selected terminal filter UAF42 low-pass filter, the cut-off frequency of the low-pass filter was adjusted to be very low at the beginning of the experiment, and the quality influence of the filter was not considered. The ripple attenuation in the passband of the low-pass filter was ignored, so the amplitude and frequency change of the particle signal when passing through the filtering and amplifying circuit in the detection circuit was not considered. As shown in Figure 15, the characteristic curves of Chebyshev-type low-pass filter vary in trend for different component parameters. The low-pass filter produces different amounts of equal ripple undulations $d^{\prime }$, $d^{\prime \prime }$ in the low frequency passband.

Low-pass filters designed with different component parameters have different characteristic curves. When the ripple in the passband of the filter is large, the amplitude gain of the output signal fluctuates greatly due to the change of the signal frequency, as shown in Figure 15a; When the ripple in the passband of the filter is small, the amplitude gain of the output signal fluctuates less due to the change of the signal frequency, as shown in Figure 15b. Therefore, it is considered that as the detection velocity increases, the signal frequency component changes, resulting in a change in the output signal gain amplitude of the detection circuit.

**Figure 16:**
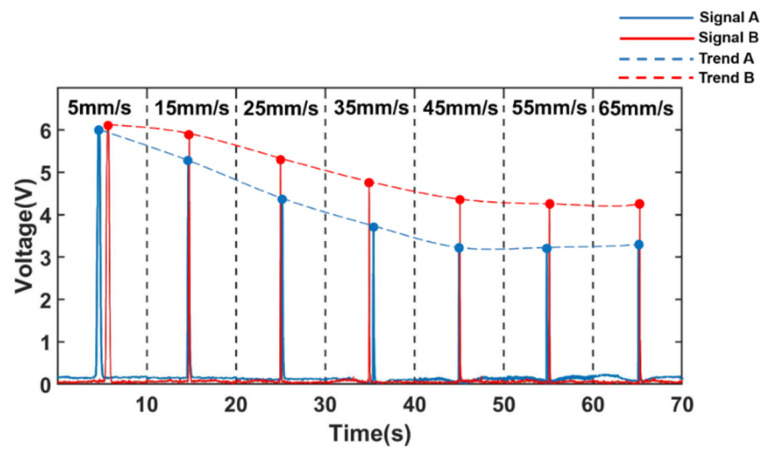
Signal change of ferromagnetic metal particles (350 μm Fe) with velocity change after adjusting the detection circuit.

**Figure 17:**
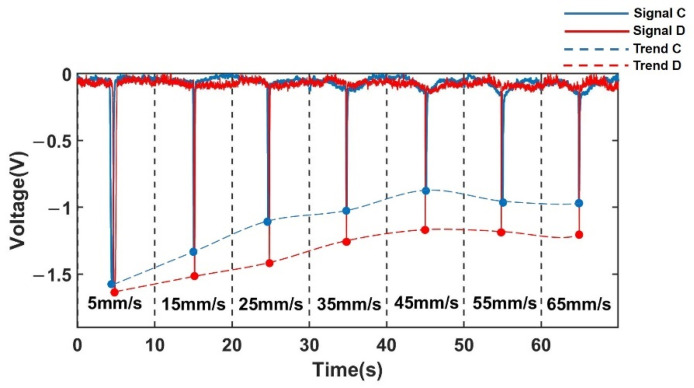
Signal change of non-ferromagnetic metal particles (350 μm Cu) with velocity change after adjusting the detection circuit.

Based on the above hypothesis, the experiment was further designed. By re-adjusting the parameters of the low-pass filter element, the amplitude-frequency characteristics [37] of the low-pass filter circuit were changed, and the response of the circuit to the signal frequency component that changes with the velocity was changed. The two potentiometers $R_{4}$ and $R_{8}$ resistors of the low-pass filter were adjusted appropriately to change its quality factor; the two potentiometers $R_{6}$ and $R_{9}$ resistors of the low-pass filter were adjusted appropriately to change its low-pass cut-off frequency. The experimental results for the detection of 350 μm iron particles and 350 μm copper particles are shown in Figure 16 and Figure 17, respectively. In Figure 16, A and B both represent 350 μm iron particles. Signal A is the voltage signal of 350 μm iron particles before adjusting the detection circuit, which is represented by the blue solid line; Trend A is the signal trend with increasing velocity, which is represented by the blue dashed line. Signal B is the voltage signal of 350 μm iron particles after adjusting the detection circuit, which is represented by the red solid line; Trend B is the signal trend with increasing velocity, which is represented by the red dashed line. In Figure 17, C and D both represent 350 μm copper particles. Signal C is the voltage signal of 350 μm copper particles before adjusting the detection circuit, which is represented by the blue solid line; Trend C is the signal trend with increasing velocity, which is represented by the blue dashed line; Signal D is the voltage signal of 350 μm copper particles after adjusting the detection circuit, which is represented by the red solid line; Trend D is the signal trend with increasing velocity, which is represented by the red dashed line.

It can be observed through two sets of experiments that after adjusting the detection circuit, the attenuation of the signal becomes slower.

Compared to the signal change amplitude of metal particles with velocity change before the adjustment of the detection circuit, the signal amplitude attenuation of iron particles was reduced from 2.6838 V to 1.8582 V, when the metal particle velocity was increased from 5 mm/s to 65 mm/s after a slight adjustment to the detection circuit, as shown in Figure 16, and the signal amplitude attenuation was reduced by 0.8256 V. As shown in Figure 17, the signal amplitude attenuation of copper particles was reduced from 0.7469 V to 0.5097 V, and the signal amplitude attenuation was reduced by 0.2372 V. This shows that the attenuation of the total signal is indeed affected by the filter circuit. Similarly, the amplifying ability of the amplifying circuit to each frequency component is also different. It is inferred that the attenuation of the total signal amplitude is indeed influenced by the amplitude-frequency characteristics in the filtering and amplifying circuits in the detection circuit.

Another reason for the analysis of the total signal attenuation is that the experimental setup of the stepper motor itself has a high current, generating non-negligible electromagnetic interference, which leads to the enhancement of low-level noise and the weakening of high-level signals through radiation interference, ultimately leading to the attenuation of the detection signal amplitude, but the influencing factor is the effect on the overall frequency component, independent of the particle velocity.

The experimental results show that in a time-harmonic magnetic field, an increase in particle velocity increases the eddy current effect in the magnetic field, resulting in a decrease in signal amplitude for ferromagnetic metal particles and an increase in signal amplitude for non-ferromagnetic metal particles. Simultaneously, the quality of the detection circuit has an impact on the signals of particles with different velocities, and the design of the detection circuit should be optimized to improve the stability of the output signal in the passband.

## 4. Conclusions

We analyzed and summarized the theory and law of the electromagnetic field for inductance differential detection, deduced the formula that causes the voltage difference of the inductive bridge to change, and optimized the subsequent processing circuit. The designed sensor can achieve low cost and portability under the premise of ensuring higher sensitivity. Based on the inductive bridge, this paper draws the following conclusions by studying the effect of velocity on particles of different materials.

In terms of the sensitive influence of the coil quality factor on the inductive bridge: Through the simulation and analysis of the equivalent circuit of the inductive bridge, it is concluded that the higher the quality factor of the coil, the higher the detection sensitivity of the inductive bridge. The quality factor of the coil is mainly affected by N, w, d. To improve the detection sensitivity of the inductive bridge, it is possible to increase the coil diameter a and reduce w and d, and appropriately increase N. Based on this method, suitable sensing and reference coils are selected for the inductive bridge.

In terms of the effect of velocity on the electromagnetic field: In the alternating magnetic field, the general law that velocity affects the inductance signal was obtained, which was verified by theoretical analysis and experimental phenomena in the magnetic field. The increased velocity will enhance the eddy current effect of the particles in the magnetic field, which will further increase the signal of non-ferromagnetic metal particles and weaken the signal of ferromagnetic metal particles.

In terms of the effect of velocity on the processing circuit: We assumed that the velocity also affects the output signal of the processing circuit, due to the abnormal weakening of the detected signal. The conclusion is that the optimized processing circuit can suppress the instability of the output signal, which arises due to the variation of the particle velocity.

This research is helpful to the development of oil online monitoring technology, which starts from the characteristics of high flow rate and high flux of oil in the mechanical systems. Future work will concentrate on optimizing the design of the detection circuit to stabilize the particle output signal at different velocities in the circuit. In addition, combined with the effect of speed on magnetic fields and circuits, we will find a balance point in the contradiction between the effect of velocity on ferromagnetic and non-ferromagnetic metals.

## Data Availability

Not applicable.
